# Rigid Plastic‐Free Fibreboards Made from “Hairy” Cellulose Fibres and Oil Palm Empty Fruit Bunch

**DOI:** 10.1002/cssc.202401878

**Published:** 2024-12-17

**Authors:** Dharu Feby Smaradhana, Diego Freire Ordóñez, Koon‐Yang Lee

**Affiliations:** ^1^ Department of Aeronautics Imperial College London Exhibition Road London SW7 2AZ UK; ^2^ Department of Mechanical Engineering Universitas Sebelas Maret Ir Sutami Surakarta Indonesia; ^3^ Department of Chemical Engineering Imperial College London Exhibition Road London SW7 2AZ UK; ^4^ Institute Molecular Science and Engineering Imperial College London Exhibition Road London SW7 2AZ UK

**Keywords:** Palm oil waste, Empty fruit bunch, Cellulose, Fibreboard, LCA

## Abstract

Empty fruit bunch (EFB), an abundant lignocellulosic residue from the palm oil milling process, is typically discarded on open land or used as mulch. In this work, a simple method that mimics a papermaking process, was developed to upcycle EFB into higher value fibreboard without the need for any polymeric binders. The cellulose network from pulp fibres was utilised to hold the otherwise loose EFB fibres together to produce a rigid EFB fibreboard. Mechanical refinement was performed using a re‐circulating colloid mill to improve the binding performance of the cellulose fibres . EFB fibreboard containing 30 wt.% of 30 min refined “hairy” cellulose fibres possessed a flexural modulus of ~2.9 GPa and strength of ~22 MPa, comparable to commercial particleboard (PB) and medium density fibreboard (MDF). A lifecycle analysis (LCA) model using ReCiPe 2016 method and Ecoinvent database was used to compare the environmental impact of the EFB fibreboard and MDF production. The results show that the EFB fibreboard possessed a lower environmental impact on global warming potential and the various end‐point impact categories compared to MDF. This work unveils new opportunities to convert palm oil waste into all‐lignocellulosic fibreboard, moving away from traditional practices which align with the concept of a circular bioeconomy.

## Graphical abstract

Empty fruit bunch (EFB), a lignocellulosic residue from the palm oil milling process often discarded or mulched, can be upcycled into plastic‐free fibreboard using “hairy” cellulose fibres as a binder. This sustainable alternative produces truly green fibreboard with mechanical properties comparable to particleboard and MDF, supporting a circular economy.

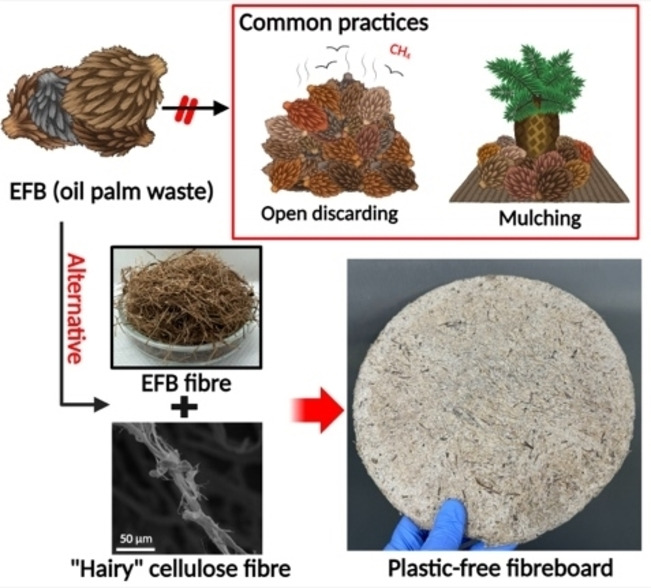

## Introduction

The global production of palm oil has drawn considerable criticism due to problematic practices, such as widespread deforestation and the burning of rainforests to make way for oil palm plantations.^[1]^ However, numerous research has demonstrated that when managed properly, the environmental impact of palm oil in the context of global warming potential, land utilisation, and water use, are at par or if not even better than rapeseed oil and soybean oil.[^2^, ^3^] In terms of carbon storage potential, one hectare of oil palm holds approximately 136.6 tons of carbon, compared to just 99.4 tons for the same area of rapeseed.^[2]^ Palm oil also has the lowest production cost compared to other vegetable oils.^[4]^ Furthermore, oil palm is considered as the most efficient crop for vegetable oil production, yielding about 4 tons of oil per hectare, which is significantly higher than other crops such as rapeseed, sunflower, and soybean that yield only 0.5 tons of oil per hectare.^[3]^

A unique feature of palm oil is the balanced mix of saturated and unsaturated fatty acids; the saturated fats provide firmness, while the unsaturated fats prevent full solidification, creating a naturally semi‐solid texture akin to butter.^[5]^ This texture is crucial because it offers stability and desirable texture, making palm oil ideal for products like margarines and shortenings.^[6]^ To attain such semi‐solid texture in alternative vegetable oils, such as soybean oil and sunflower oil, a hydrogenation step needs to be performed prior to use.^[7]^ Palm oil′s high melting point and excellent oxidation resistance also leads to a longer shelf life, further enhancing its versatility in a wide range of applications.^[8]^ As a result, palm oil‐derived products can be found in 50 % of the world′s packaged supermarket products and is also used as sustainably derived biofuel.^[9]^ A recent report by European Commission projected an increase in palm oil demand to about 142 million metric tons by 2050.^[10]^

A critical issue that has not been adequately addressed in the oil palm industry, however, is the management of residue biomass waste from palm oil mill. The extraction of palm oil involves sorting and sterilizing fresh fruit bunches (FFBs) before pressing the fruit mesocarp to extract crude palm oil (CPO).^[11]^ Generally speaking, the production of 1 ton of CPO generates 1.1 tons of empty fruit bunches (EFBs), 0.68 tons of palm pressed fibres (PPF), and 0.32 tons of palm kernel shells (PKS).^[12]^ Due to the low moisture content, minimal ash content, and high energy values, PKS and PPF are typically used as fuel in the boilers of the palm oil mill.^[13]^ EFB, which has a composition of 43–65 % cellulose, 17–34 % hemicellulose and 13–25 % lignin,^[14]^ is derived from the leftover parts of FFB after the fruits have been removed.Unfortunately, EFB is not suitable as fuel due to its high moisture content (*ca*. 60 %).^[12]^ In 2023, approximately 87 million tons of EFB were generated globally.

The end‐of‐life option for EFB was predominantly incineration a few decades ago and this led to an escalation in greenhouse gas emissions.^[15]^ With the tightening of environmental regulation and the recognition of their nutritional profile, EFB is frequently used as mulch within oil palm plantations in Indonesia.^[16]^ Yet, this approach is only implemented by large industrial estates, which cover only 48 % of Indonesia′s palm oil landscape.^[17]^ The remaining land is managed by smallholder farmers, who often adopt the simpler end‐of‐life method of discarding the EFB in large piles on open area,^[17]^ which leads to an increase in methane emission.^[18]^ The adoption of this end‐of‐life method by smallholder famers is due to the labour‐intensive handling and transport expenses of EFB, as well as the lack of economic incentive.^[19]^ In addition, mulching EFB can only boost the fruit production of oil palm trees by up to 5.9 % over a 15 year span due to the slow release of nutrients.[^15^, ^17^] Immediate‐acting mineral fertilisers are more appealing to smallholder farmers wanting swift outcomes.^[17]^ The high organic content of EFB also raises concerns about pest attraction.^[20]^ Therefore, exploring alternative uses for EFB is crucial.

In this study, we report the upcycling of EFB fibres into rigid and plastic‐free fibreboards using only cellulose pulp as the binder. Such fully biobased fibreboard can serve as an alternative to medium density fibreboard (MDF) and particleboard (PB), which are manufactured by binding wood chips or fibres with up to 10 wt.% of formaldehyde‐based resins.^[21]^ As the market price of EFB and cellulose pulp are approximately $12/ton^[22]^ and $540/ton^[23]^ respectively, transforming EFB into high‐value products like fibreboards that could be sold at a higher price not only fosters greater sustainability but also supports the economic resilience of both the agricultural and industrial sectors. The manufacturing process of these EFB fibreboards mimicked a papermaking method following our previous work.[^24^, ^25^] To enhance the binding between cellulose pulp fibres, a mechanical refinement process utilising a recirculating colloid mill was applied before mixing them with EFB fibres. The refinement process led to the creation of “hairy” cellulose fibres, *i. e*., cellulose fibres with microfibrils still partially attached on the surface of the primary fibres. This process increased entanglement and contact area between the hairy cellulose fibres itself, as well as between the hairy cellulose fibres and the EFB fibres. Consequently, the EFB fibreboards using “hairy” cellulose fibres as the binder demonstrated higher mechanical performance compared to those using unrefined cellulose fibres as the binder. Additionally, a lifecycle assessment (LCA) using ReCiPe 2016 also confirmed that the production of the EFB fibreboard is more environmentally sustainable than MDF.

## Results and Discussion

### Fabrication of EFB Fibreboards

**Figure 1:**
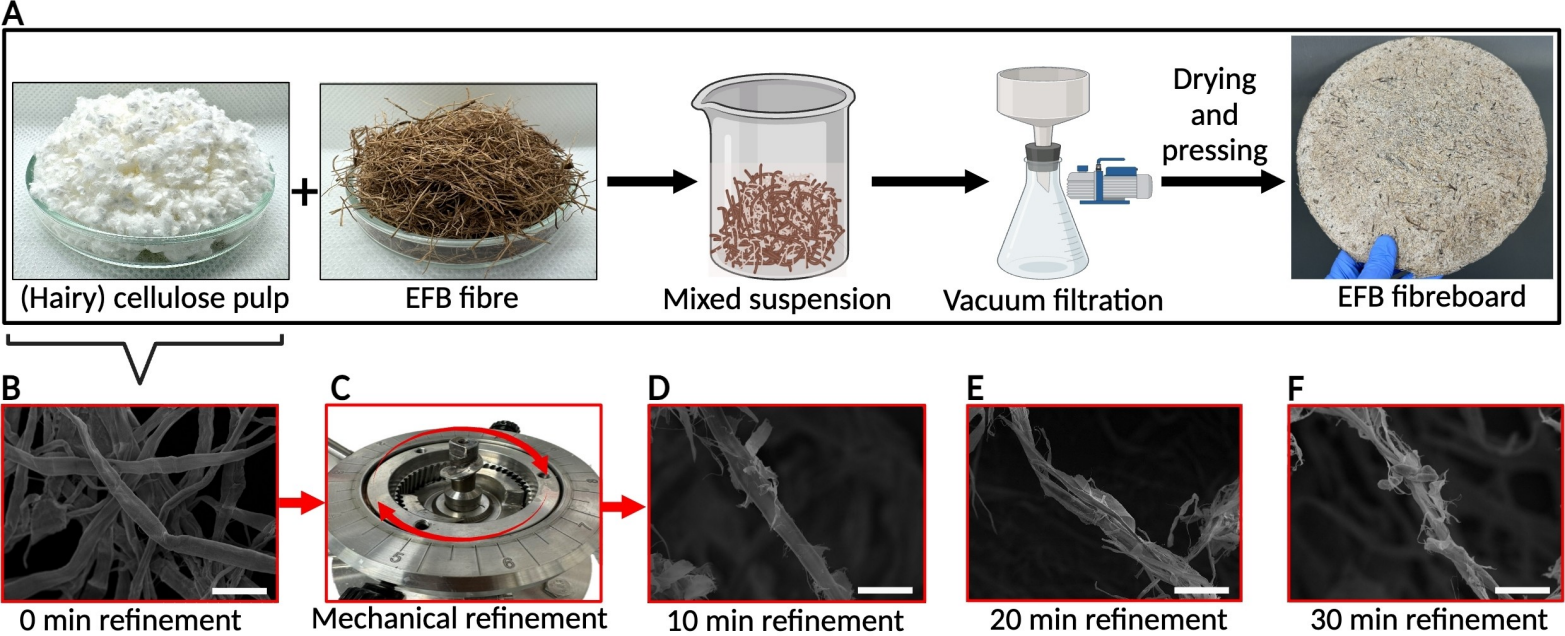
(A) Manufacturing process of EFB fibreboards using (hairy) cellulose fibres as binder. Effect of mechanical refinement on the microstructures of (hairy) cellulose fibres: (B) unrefined cellulose fibre, (C) recirculating colloid mill, (D) 10 min refined hairy cellulose fibre, (E) 20 min refined hairy cellulose fibre and (F) 30 min refined hairy cellulose fibre. Scale bar: 50 μm. Illustrations in Figure 1A were created with BioRender.com.

The manufacturing rigid and plastic‐free EFB fibreboard utilising cellulose pulp as the binder is shown schematically in Figure 1A. Briefly, EFB fibres of *ca*. 10 mm in length were mixed into an aqueous cellulose pulp suspension consisting of neat or hairy cellulose pulp fibres at a combined slurry consistency of 25 g/L. Hairy cellulose pulp fibres were produced by refining neat pulp using a re‐circulating colloid mill following a previously developed protocol.^[26]^ Three different refining time, namely 10, 20 and 30 min, were used. The morphology of hairy cellulose fibres is shown in Figure 1. The EFB/(hairy) cellulose fibre suspension was then dewatered using vacuum‐driven filtration, followed by press‐drying under a weight of 2 t at 120 °C for 1 h. All EFB fibreboards reported in this work possessed a grammage of 2000 g m^−2^. A detailed description of the manufacturing of the EFB fibreboards, as well as the details of the different characterisations can be found in ESI S1. It is worth mentioning at this point that without any cellulose fibres, an EFB fibreboard cannot be produced as the EFB fibres are loosely held together by friction.

### Structure and Morphology of EFB Fibreboards

Figure 1B shows the morphology of neat cellulose fibres, which possess a smooth surface and a fibre width of *ca*. 25 μm. By passing the cellulose pulp through the re‐circulating colloid mill (Figure 1C) for different amount of time, different degree of fibrillation can be achieved. Refining the cellulose fibres for 10 min in a re‐circulating colloid mill unravel some of the surface cellulose microfibrils, producing hairy fibres (Figure 1D). A further increase in refining time to 20 min (Figure 1E) and 30 min (Figure 1F) leads to a higher degree of fibrillation along with pulp fibre splitting. During the refining process, the cellulose fibres are forced through the gap between the rotor and the stator discs (~0.1 mm). As both discs contain a toothed surface (see Figure 1C), the efficacy of fibrillation is expected to be higher than a conventional PFI mill, which has a toothed surface and a smooth surface configuration,^[27]^ but less than a stone grinder typically used to produce cellulose nanofibrils (Masuko Supermasscolloider for example), which has a very small absolute gap size (~0.01 mm size).^[28]^ Consequently, the shearing action only affected the surface of the cellulose fibres in our work, resulting in the observed surface fibrillation. It is worth mentioning at this point that in addition to such top‐down approach, hairy cellulose fibres can also be produced via a bottom‐up approach by attaching bacterial cellulose nanofibrils onto a larger, primary cellulose fibre.[^29^, ^30^]

**Figure 2:**
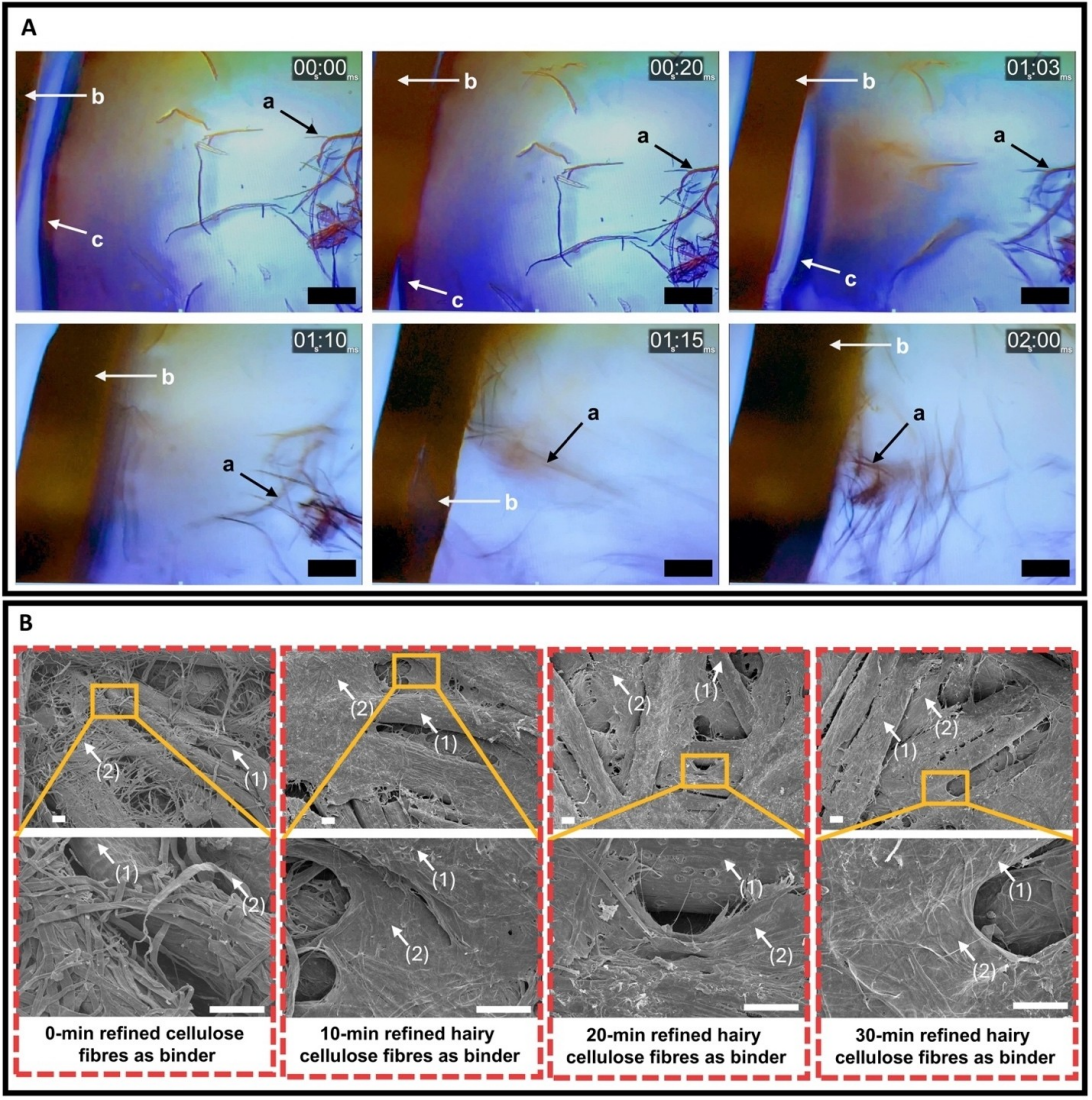
(A) Still video frames of a single EFB fibre immersed in a droplet of pulp suspension. (B) Representative scanning electron micrograph of EFB fibreboards containing 10 wt.% (hairy) cellulose fibres as binder. Labels (a), (b) and (c) denote agglomeration of (hairy) cellulose fibres, EFB fibres and droplet edge, respectively. Labels (1) and (2) denote EFB fibre and (hairy) cellulose fibre, respectively. Scale bar: 100 μm.

EFB fibres are hygroscopic^[31]^ and contain lumens of *ca*. 10 μm in diameter.^[32]^ Therefore, when EFB fibres are immersed in a (hairy) cellulose pulp suspension, water absorption through capillary action occurs.^[14]^ This then causes the previously suspended (hairy) cellulose fibres to flow and eventually attaching themselves onto the surface of the EFB fibres, as they are too large to penetrate into the lumen. Such flow‐and‐attach effect is evident from the still video frames of a single dry EFB fibre immersed in a droplet of pulp suspension (Figure 2A). An agglomerate of cellulose fibres (label [a] in Figure 2A) can be seen moving towards the surface of the EFB fibre within a second after initial contact with the pulp suspension. The full video of this flow‐and‐attach effect can be found in ESI. Upon dewatering and wet pressing, the (hairy) cellulose pulp fibres that were coated on the EFB fibres are in contact with each other. As the drying of the wet pressed filter cake progresses, inter (hairy) cellulose fibre bonding via van der Waals’ interaction, hydrogen bonding and fibre/molecular entanglement formed, binding the EFB fibres together (see Figure 2B for exemplarily scanning electron micrographs of EFB fibreboards containing 10 wt.% of cellulose fibre as the binder). Since the drying process was also conducted under restraint, lateral shrinkage of the wet pressed filter cake was prevented. This further removes slackness and activating^[33]^ the bonded (hairy) cellulose fibre network, increasing the rigidity of the resulting EFB fibreboard. Simple weight gain measurement estimated the loading fraction of (hairy) cellulose pulp fibres on single EFB fibre was ca. 15 wt.–%.

**Figure 3:**
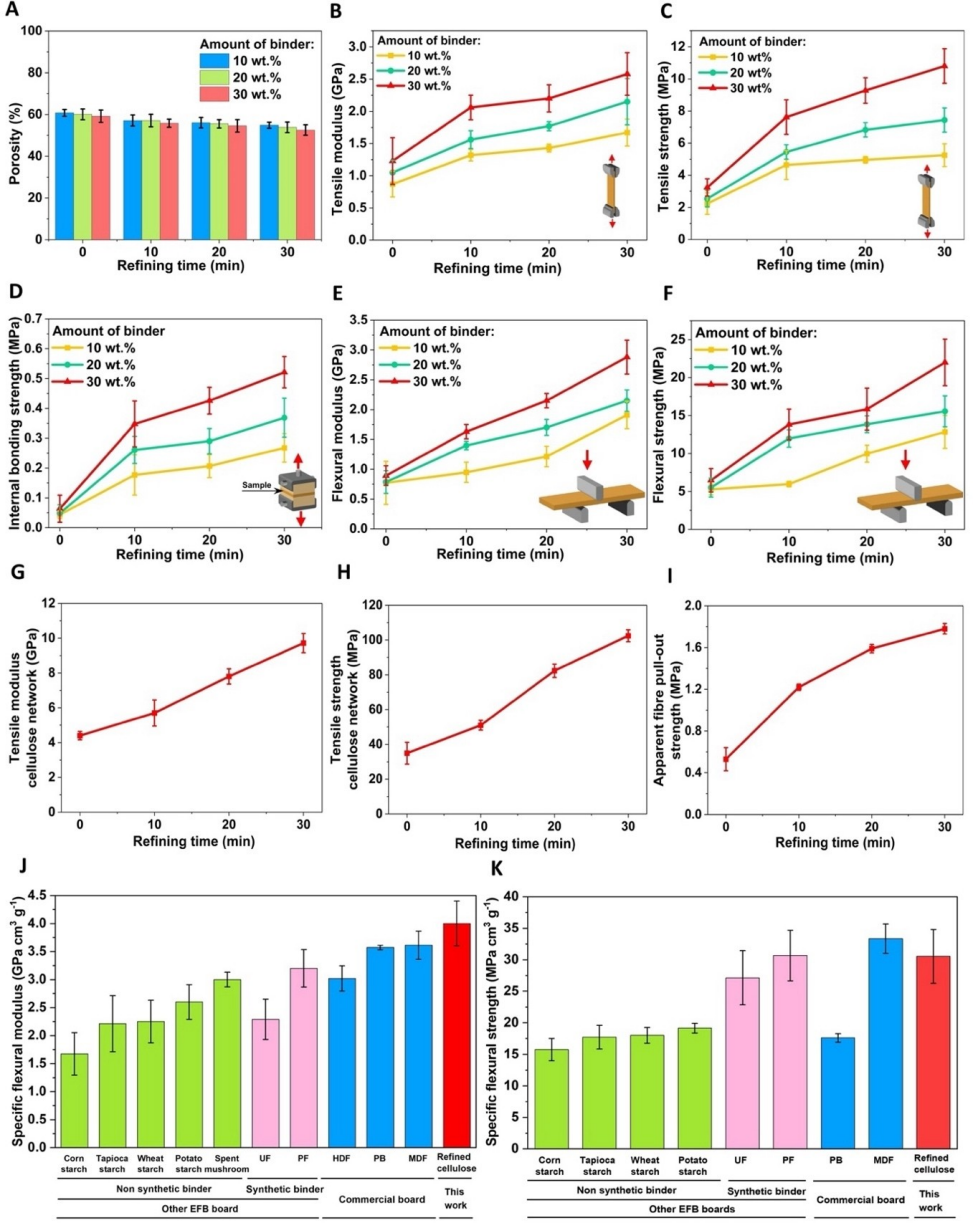
Physical and mechanical of EFB fibreboards utilising (hairy) cellulose fibres as binder: (A) porosity, (B) tensile modulus and (C) strength, (D) internal bonding strength, (E) flexural modulus and (F) strength. (G) Tensile modulus and (H) strength of (hairy) cellulose fibre network. (I) Apparent EFB fibre pull‐out strength from (hairy) cellulose fibre network. Comparison of (J) specific flexural modulus and (K) strength of EFB fibreboard with 30 wt.% 30 min‐refined “hairy” cellulose fibres as binder to other EFB boards using different types of binders and commercial fibreboards.[^34^, ^35^, ^36^, ^37^, ^38^] UF, PF, PB, HDF and MDF denote urea formaldehyde, phenolic formaldehyde, particleboard, high density fibreboard and medium density fibreboard, respectively. Error bars indicate standard deviation.

The porosity of the EFB fibreboards was found to be independent of both the amount of cellulose binder and the refining time of the cellulose binder used (Figure 3A). The porosity values for the EFB fibreboards remained within the range of 53 %–61 %, indicating that variations in binder loadings and refining duration did not significantly impact the porosity.

### Mechanical Properties of EFB Fibreboards

Figure 3B and C summarise the tensile properties of the different EFB fibreboards manufactured. Increasing the (hairy) cellulose fibre binder content and cellulose pulp refining time improve the tensile properties of the resulting EFB fibreboards. At a binder content of 10 wt.%, the EFB fibreboard with unrefined cellulose fibres exhibited a tensile modulus and strength of only 0.9 GPa and 2 MPa, respectively. When 10 min refined hairy cellulose fibre was used as the binder, both the tensile modulus and strength improved, with the tensile modulus increasing by 52 % to 1.3 GPa and the strength doubled to 4.6 MPa. Increasing the refining time of the cellulose pulp binder to 30 min increased the tensile modulus to 1.7 GPa and the tensile strength to 5.3 MPa. A similar trend was also observed for higher cellulose fibre binder loading. At 20 wt.% loading of 30 min refined hairy cellulose fibres as the binder, the tensile modulus and strength of the EFB fibreboard were measured to be 2.2 GPa and 7.4 MPa, respectively. At 30 wt.% loading with the same refining time for hairy cellulose fibres as the binder, the tensile modulus and strength increased to 2.6 GPa and 10.8 MPa, respectively.

We also investigated the internal bonding strength (Figure 3D) and the flexural properties (Figure 3 E and F) of our EFB fibreboards. Trends similar to the tensile properties were observed; an increase in the loading of (hairy) cellulose fibre binder increases the internal bonding strength and the flexural performance. The longer the refining time of the (hairy) cellulose fibre binder used, the better the internal bonding strength and flexural properties. The internal bond strength of the EFB fibreboard with 10 wt.% cellulose fibres as the binder significantly improved from 0.04 MPa for unrefined cellulose fibre binder to 0.27 MPa for 30 min refined hairy cellulose fibre binder. When the binder content was raised to 30 wt.%, 30 min refinement of hairy cellulose fibres as the binder further enhanced the internal bond strength of the EFB fibreboard to 0.52 MPa, representing an eightfold increase over the EFB fibreboard with the same amount of unrefined cellulose fibres as the binder. In terms of flexural properties, the modulus and strength of the EFB fibreboard with 10 wt.% unrefined cellulose fibres as the binder were measured to be 0.8 GPa and 5.3 MPa, respectively. When 30 min refined hairy cellulose fibres were used as the binder, both flexural modulus and strength increased to 1.9 GPa and 12.8 MPa, respectively. At 30 wt.% loading of 30 min refined hairy cellulose fibres as the binder, the flexural modulus of the EFB fibreboard rose more than double to 2.9 GPa, and the flexural strength by 240 %, reaching 22 MPa, compared to the EFB fibreboard with the same amount unrefined cellulose fibres as the binder.

The increase in the mechanical performance of the EFB fibreboards with increasing refining time of the hairy cellulose fibres stems from the increase in the tensile properties of the hairy cellulose fibre network (in the form of a paper sheet) (see Figure 3G and H). Without refinement, the resulting cellulose fibre network possessed a tensile modulus of only 4.4 GPa and a strength of 35 MPa. Increasing the refining time to 10 min resulted in the improvement in the tensile modulus and strength of the resulting hairy cellulose fibre network to 5.7 GPa and 51 MPa, respectively. The tensile modulus of the hairy cellulose fibre network refined for 30 min showed a more notable improvement to 9.7 GPa; an increase of 121 % over unrefined cellulose fibre network. A similar trend was also observed for the strength of the hairy cellulose fibre network refined for 30 min which indicated an increase by 198 %–102 MPa compared to unrefined cellulose fibre network. This improvement can be attributed to the presence of hairy cellulose fibres which lead to a denser fibre network provided by a larger contact area for mechanical entanglement and inter fibre bonding.

We also investigated the apparent EFB fibre pull‐out strength from a (hairy) cellulose fibre network (Figure 3I). To evaluate this, we embedded single EFB fibres into a wet filter cake made from unrefined and refined cellulose pulp, followed by press‐drying at 120 °C. The measured apparent pull‐out strength of the single EFB fibres embedded in the unrefined cellulose fibre network was 0.53 MPa. When the EFB fibres were embedded in hairy cellulose fibres, the apparent pull‐out strength increased with increasing cellulose pulp refining time. Specifically, the pull‐out strength for EFB fibres from 10, 20, and 30 min refined cellulose fibres were 1.2 MPa, 1.6 MPa and 1.8 MPa, respectively. These results corroborate with the mechanical performance of EFB fibreboards reported in Figure 3B–F and confirms that the higher the degree of fibrillation of the hairy cellulose fibres, the better the bonding between the EFB fibre and the hairy cellulose fibres.

Some authors have also reported the mechanical properties of EFB fibreboards using different types of bio‐based binders, including various starches^[34]^ and spent mushroom^[35]^ as well as formaldehyde binders, including urea formaldehyde (UF)^[36]^ and phenol formaldehyde (PF).^[38]^ We therefore benchmarked the flexural properties of our best performing EFB fibreboard (30 wt.% hairy cellulose fibre refined for 30 min as the binder) with these literature data, as well as commercial high density fibreboard (HDF),^[37]^ medium density fibreboard (MDF) and particleboard (PB) (see Figure 3J and K). Our best performing rigid and plastic‐free EFB fibreboard possesses superior specific flexural modulus compared to previously reported EFB fibreboards using other bio‐based and formaldehyde‐based binders, as well as commercial fibreboards. The specific flexural strength of our best performing EFB fibreboard is also higher than that of previously reported EFB fibreboards and commercial PB.

### Lifecycle Assessment (LCA)

**Figure 4:**
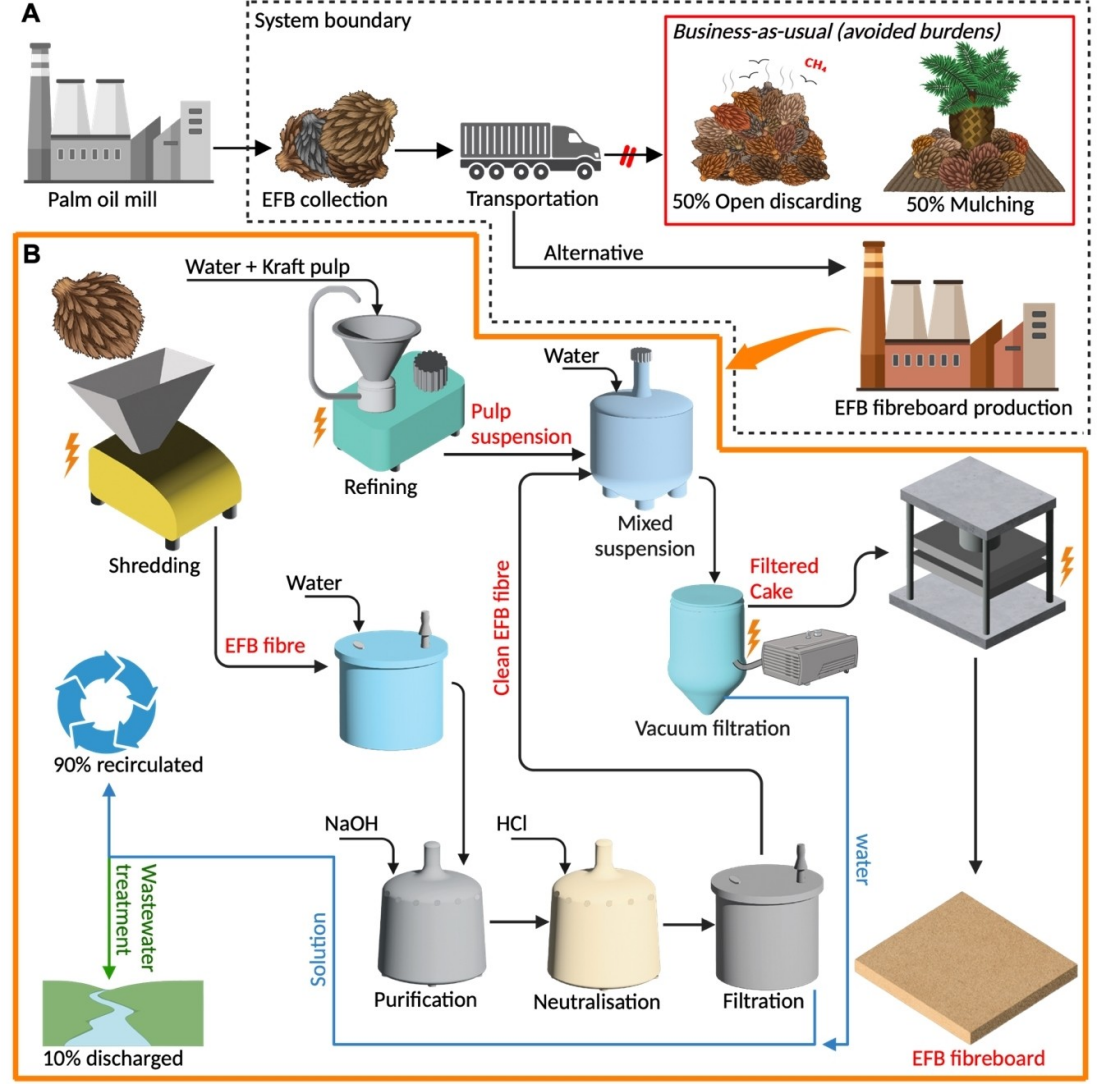
Cradle‐to‐gate analysis for the production of EFB fibreboard utilising hairy cellulose fibres as binder: (A) system boundary of EFB fibreboard production and (B) process flowsheet model of EFB fibreboard production for lifecycle analysis. Illustrations were created with BioRender.com.

The previous sections demonstrate that hairy cellulose fibres can be used to upcycle EFB into high performance materials. This will create a stronger demand for EFB that is destined to be discarded and mulched to be used in selected engineering applications. To investigate whether the manufacturing of EFB fibreboards using hairy cellulose fibres as binder is also sustainable, the environmental impact of the production of the EFB fibreboard was examined using LCA following EN ISO 14040.^[39]^ All calculations were carried out using SimaPro version 9.5 integrated with the Ecoinvent 3.8 database based on the ReCiPe 2016 method.^[40]^ A cradle‐to‐gate approach was developed to include all lifecycle emissions from waste collection (Figure 4A) to the industrial‐scale production of EFB fibreboards (Figure 4B). MDF was used as our benchmark for comparison in our LCA model and its lifecycle inventory is obtained from literature.^[41]^ Both the MDF and the EFB fibreboard are assumed to have the same durability. To compare materials with different mechanical performance, a performance indicator based on the specific flexural modulus was employed. This indicator calculates the mass of the functional unit ($m_{f.u.}$ ) (our benchmark MDF) required to achieve the same level bending rigidity as the best performing EFB fibreboard made from 1 kg of raw EFB (containing a dry matter of 40 %) and 30 wt % hairy cellulose fibres refined for 30 min as the binder. The formula $m_{f.u.}$ is provided in the Equation 1 :

(1) $$m_{f.u.}\;=m_{ref}\;\times {\left[\frac{E_{ref}}{E_{f.u.}}\right]}^{1/3}\left[\frac{\rho_{f.u.}}{\rho_{ref}}\right]$$

where $m_{ref}$ , $E_{ref}$ , and $\rho_{ref}$ are the mass, flexural modulus and density of the EFB fibreboard, respectively. The terms $E_{f.u.}$ and $\rho_{f.u.}$ correspond to the flexural modulus and density of the MDF counterpart, respectively. The derivation of Equation (1) can be found in the literature.^[42]^ Our LCA model utilises Indonesia′s high voltage electricity grid as its primary energy source, which comprises of 53 % lignite, 21 % combined cycle gas, 8 % hydro, 5 % natural gas, 5 % oil, 3.5 % biomass, and other sources.^[43]^ This energy grid was chosen as Indonesia is the largest exporter of palm oil. Our study focuses on the global warming potential (GWP) at the midpoint and the three endpoint categories, *i. e*., human health (HH), ecosystems quality (EQ), and resources scarcity (RS). Detailed information about the LCA method, including the inventory data and assumptions can be found in ESI S2.

**Figure 5:**
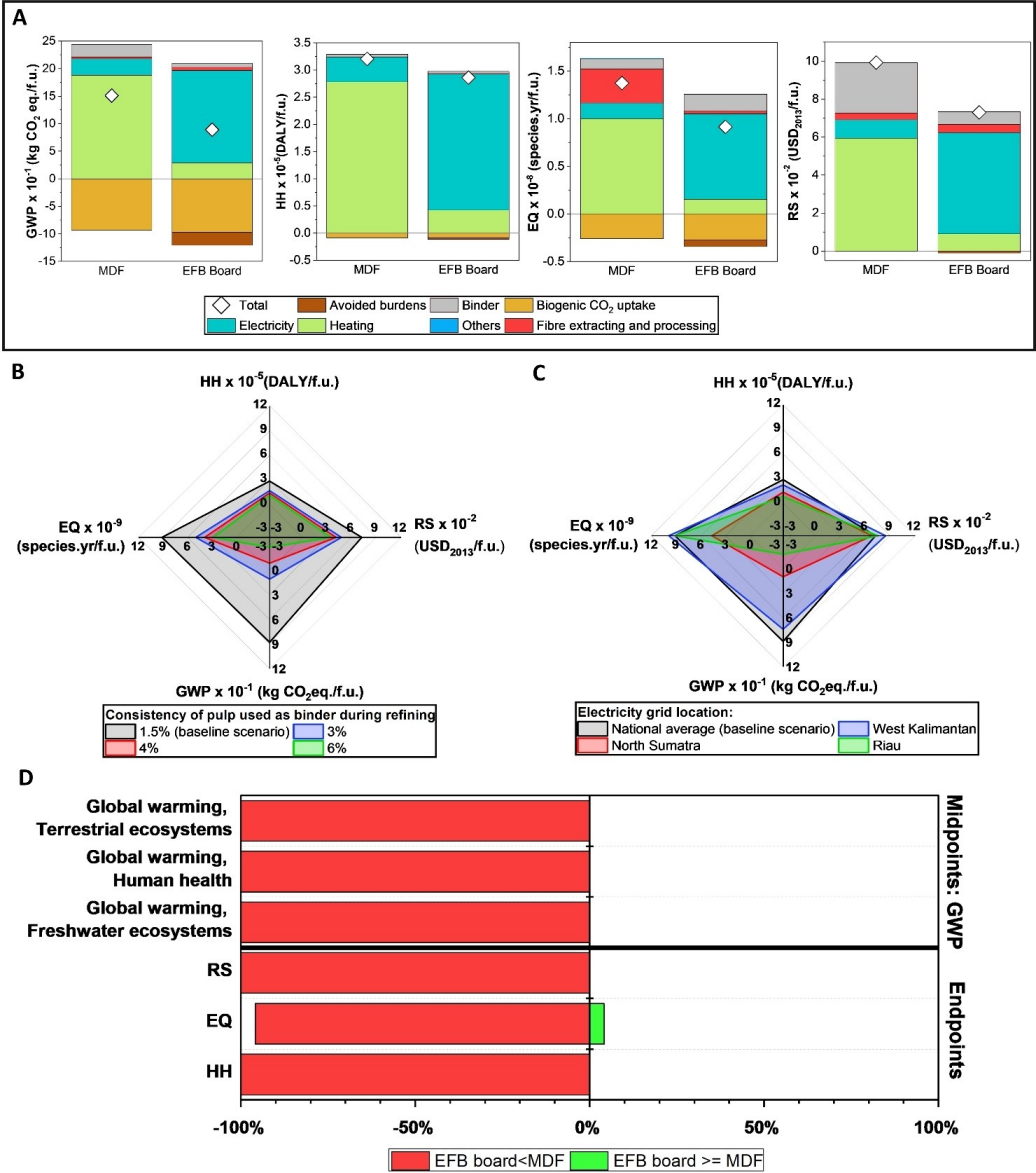
(A) ReCiPe 2016 LCA results for the EFB fibreboard and MDF productions. Sensitivity analysis on the production of EFB fibreboards: (B) cellulose pulp consistency during refining and (C) the electricity grid location based on three different provinces in Indonesia. (D) ReCiPe 2016 relative environmental uncertainty. “Others“ include direct emission, water and wastewater. Biogenic CO_2_ uptake is calculated based on 50 % of the carbon content in the biomass weight. Avoided burdens represent environmental credits that are derived from circumventing the environmental impacts associated with a business‐as‐usual scenario, with detailed parameters and inventories available in ESI S2.

Figure 5A shows the GWP and the three endpoint‐level categories for the production of MDF and our best performing EFB fibreboard. We accounted for biogenic CO_2_ uptake in our LCA by considering the biomass content of materials such as EFB and wood pulp in EFB fibreboards and wood fibres in MDF, based on the premise that 50 % of the biomass′s dry weight is carbon.^[44]^ This consideration granted environmental credits to EFB fibreboard and MDF, as the biomass can act as a carbon sink during their lifetime.^[45]^ It resulted in credits for GWP, HH, and EQ of −0.93 kgCO_2_e, −8.62×10^−7^ DALY and −0.26×10^−8^ species.yr per functional unit for MDF and −0.97 kgCO_2_e, −9×10^−7^ DALY and −0.27×10^−8^ species.yr per functional unit for the EFB fibreboard, respectively. The production of our best performing EFB fibreboard has a lower environmental impact than the production of MDF across the GWP and the three endpoint categories. The net GWP, HH, EQ, and RS for the EFB fibreboard production are 0.89 kgCO_2_e, 2.86×10^−5^ DALY, 0.91×10^−8^ species.yr and 7.3×10^−2^ USD_2013_, respectively, compared to 1.51 kgCO_2_e, 3.21×10^−5^ DALY, 1.37×10^−8^ species.yr and 9.92×10^−2^ USD_2013_ for the MDF production. The lower impact can be attributed to the avoidance of the “business‐as‐usual” scenario (see Figure 4A), where 50 % of the total EFB produced would otherwise be mulched and the remaining would be landfilled in an open area. The latter can lead to a methane emission of around 26.7 kg/ton of EFB. Avoiding the practice of open discarding has a significant benefit, reducing GWP to about 450 kgCO₂e per ton of EFB compared to the avoidance of mulching practice which only reduces GWP to about 23 kgCO₂e per ton of EFB. By diverting the EFB from these common practices, the EFB fibreboard production effectively gains credits from these avoided burdens in GWP, HH, EQ and RS of −0.24 kgCO_2_e, −2.41×10^−7^ DALY, −0.072×10^−8^ species.yr and 0.11×10^−2^ USD_2013_ per functional unit, respectively. Moreover, the EFB fibreboard benefits from a slightly higher flexural modulus compared to MDF (Figure 3G), which translates into a weight saving (and a volume saving). It is evident that in the production of both EFB fibreboard and MDF, the primary source of environmental impacts comes from the energy used for utilities, including heating and electricity for equipment. Unlike MDF, where the main hotspot stems from the heating required for the drying of wood fibres and the curing of synthetic binders such as urea formaldehyde and phenol formaldehyde that typically requires a high temperature of between 140 °C and 220 °C,[^41^, ^46^] the EFB fibreboard production is predominantly impacted by its electricity use, especially for the refining of cellulose pulp used as the binder (see ESI S3). The refining of pulp accounts for 75 %, 79 %, 67 % and 68 % of the total positive impact on GWP, HH, EQ and RS, respectively. This is because Indonesia′s national electricity grid is primarily powered by lignite, a fuel source with one of the highest environmental impacts (see ESI S4). Lignite′s high carbon content, low energy density, and emissions of potent greenhouse gases, including CO₂, CH₄, and N₂O, contribute substantially to its significant environmental impact.^[47]^ In the EQ category, alongside heating, the use of wood fibres as the primary material in MDF production creates a significant environmental impact, registering the second highest contributor at 0.36×10^−8^ species.yr per functional unit, accounting for 22 % of the total positive impact in this category. While a wood‐derived binder used in the EFB fibreboard production also contributes to the EQ impact, its effect is less pronounced (0.18×10^−8^ species.yr) because it is not the primary material. Furthermore, the use of urea formaldehyde as the binder in MDF production has a pronounced environmental impact in the RS category, amounting to 2.66×10^−2^ USD_2013_ per functional unit and contributing to 27 % of the total positive impact in this category. In contrast, the EFB fibreboards employ a plastic‐free binder, leading to a lower impact on RS.

### LCA: Sensitivity Analysis

Sensitivity analysis is conducted, focusing on two crucial aspects of the LCA results of the EFB fibreboard production: the consistency of the cellulose pulp suspension during refining to produce “hairy” cellulose fibres as binder and the electricity grid based on the provincial location of the EFB fibreboard factory in Indonesia. The former explores the environmental impact of increasing the consistency of the cellulose pulp suspension to achieve energy savings in the refining process. In the baseline scenario of the EFB fibreboard production, a cellulose pulp suspension with a consistency of 1.5 % was used, reflecting lab‐scale production in this work. Increasing the consistency of the pulp suspension can enhance the efficiency of the refining process by reducing the amount of water mixed with the pulp. This reduction in water content lowers the energy required for refining the mixture, thereby decreasing overall energy consumption.^[27]^ Our sensitivity analysis explored the effect of increasing the pulp consistency to 3 %, 4 %, and 6 % on the environmental impact of EFB fibreboard production. The results (Figure 5B) indicate that increasing pulp consistency decreases environmental impact across GWP and all the endpoint categories. Notably, a 6 % consistency of the pulp suspension achieves a GWP reduction of approximately 130 % when compared to the baseline, even reaching negative emissions (−0.28 kg CO_2_e per functional unit). Additionally, the environmental impact on HH, EQ, and RS are significantly reduced by approximately 61 %, 68 %, and 51 % respectively, reaching to 1.1×10^−5^ DALY, 2.9×10^−8^ species.yr and 3.5×10^−2^ USD_2013_, respectively. Therefore, we recommend utilising higher cellulose pulp consistency during refining when scaling up EFB fibreboard production to reduce electricity consumption and to enhance the environmental friendliness of the finished products. The variability in environmental impact from different electricity sources is prominent, as the geographic location of energy production in Indonesia significantly affects their carbon footprint. This variation is due to the diverse electricity supply across provinces, influenced by local natural resources and infrastructure.[^43^, ^48^] The potential locations for the EFB fibreboard production factory are ideally situated near oil palm plantations and palm oil mills to minimise transport costs. Riau, North Sumatra, and West Kalimantan, which are the provinces with the largest oil palm plantations in Indonesia,^[49]^ are considered the most suitable locations for establishing the EFB fibreboard production factory. The LCA of the EFB fibreboard production based on the electricity grid of these three provinces was conducted and compared to the baseline scenario, which uses national average electricity grid. Detailed electricity grid profiles for these provinces can be found in ESI S2. As shown in Figure 5C, the LCA results of the EFB fibreboard production across various Indonesian provinces demonstrate significant regional differences in overall environmental impact. Among the provinces assessed, Riau exhibits the lowest environmental impact on GWP and HH of −0.17 kgCO_2_e and 0.8×10^−5^ DALY per functional unit, respectively. This is attributed to Riau′s reliance on renewable energy sources, with 54 % of its electricity production coming from renewables, i. e., 48.4 % biomass, 1.2 % biogas, and 4.4 % hydroelectric. However, the predominance of biomass in its energy profile results in an equal environmental impact value on EQ compared to the baseline scenario. Conversely, North Sumatra shows the lowest environmental impact on EQ and RS of 4.66×10^−9^ species.yr and 6.7×10^−2^ USD_2013_ per functional unit, respectively. This is due to the high proportion of hydro (26.5 %) and deep geothermal (11.5 %) in its electricity grid profile, which are known for their lower environmental impact on EQ and RS compared to the other electricity sources (see ESI S4). Meanwhile, West Kalimantan demonstrates impacts that are comparable to the baseline scenario, with notably higher impacts on EQ and RS. This is primarily due to its heavy reliance on lignite and oil for electricity generation, which constitute 40 % and 21 % of its energy mix, respectively. Given the comprehensive LCA results across various Indonesian provinces, Riau emerges as the optimal location for establishing an EFB fibreboard factory, primarily due to its significantly lower impacts on GWP and HH, which are supported by its substantial reliance on renewable energy sources.

### LCA: Uncertainty Analysis

A Monte Carlo simulation was conducted to analyse the uncertainty within the inventory data, evaluating GWP and the endpoint indicators using SimaPro. This method involves random sampling to model data variability through a log‐normal distribution, of which, each uncertain parameter undergoing 1000 iterations with a confidence intervals of 95 %.^[50]^ The pedigree matrix, which evaluates the data on criteria such as reliability and completeness, is used to determine the parameters of the underlying log‐normal distribution that model the inventory′s elementary flows like feedstock, emissions, and waste.^[51]^ The absolute uncertainties associated with the environmental impact of MDF and EFB fibreboard production in GWP and the three endpoint categories can be seen in ESI S5. However, these results are specific to the absolute uncertainties of each product/system and should not be used for direct comparisons between different products/systems, as this limitation prevents definitive comparative conclusions.^[50]^ To facilitate comparative analysis, Figure 5D presents pairwise probability showing instances where the EFB fibreboard production has lower environmental impact than MDF based on 1000 Monte Carlo iterations. It is suggested that an alternative should outperform its counterpart in at least 90 % of the Monte Carlo samples to ensure a satisfactory level of discrimination.[^50^, ^52^] This level of discrimination is consistently met across GWP and the three endpoint categories. Therefore, the overall uncertainty analysis in this study strongly supports that the EFB fibreboard production is more environmentally friendly than the MDF.

## Conclusions

We demonstrated in this work that EFB can be upcycled into a high value fibreboard utilising cellulose pulp as the binder, removing the use of formaldehyde binders. Cellulose fibres held the loose EFB fibres together, producing a rigid EFB fibreboard. The mechanical refinement was performed to improve the binding performance of cellulose fibres. It was found that longer refining times led to a higher degree of fibrillation or hairy cellulose fibre formation, which increases the contact points among adjacent hairy cellulose fibres and between hairy cellulose fibres and EFB fibres. This led to the improvement in mechanical performance of the EFB fibreboards, *i. e*., tension, bending and internal bonding. The flexural properties of the best performing EFB fibreboard produced in this work (30 wt.% of 30 min refined hairy cellulose fibres as the binder) was found to be superior compared to the other EFB fibreboards using different types of binders as reported in the literature and even commercial fibreboards such as HDF, MDF and PB. The LCA results indicate that EFB fibreboard production has a lower environmental impact than MDF across Global Warming Potential (GWP) and the three endpoint categories: HH, EQ and RS. In the production of the EFB fibreboard, the energy consumption of mechanical refinement for producing “hairy” cellulose fibre binders is the highest contributor to the environmental impact on GWP and the endpoint categories. However, sensitivity analysis shows that this impact can be reduced by increasing the consistency of the cellulose fibre suspension during the refining process. Additionally, selecting the appropriate location for the EFB fibreboard production can further reduce environmental impact, with Riau emerging as a favourable location due to its cleaner electricity sources. The estimated cost for producing EFB fibreboards is also relatively low, approximately $124 per m^3^ based on the cost of the materials used. This work provides a sustainable alternative for the use of EFB for semi‐structural applications moving away from traditional practices such as open discarding and mulching.

## Conflict of Interests

The authors declare no conflict of interest.

1

## Supporting information

As a service to our authors and readers, this journal provides supporting information supplied by the authors. Such materials are peer reviewed and may be re‐organized for online delivery, but are not copy‐edited or typeset. Technical support issues arising from supporting information (other than missing files) should be addressed to the authors.

Supporting Information

Supporting Information

## Data Availability

The data that support the findings of this study are available from the corresponding author upon reasonable request.
