# Supplementary material for: Rigid Plastic‐Free Fibreboards Made from “Hairy” Cellulose Fibres and Oil Palm Empty Fruit Bunch
Source: ChemSusChem. 2024 Dec 17;18(8):e202401878. doi: 10.1002/cssc.202401878 (PMC11997918; doi:10.1002/cssc.202401878)
Supplement: Supplementary file 1 — Supporting Information [file CSSC-18-e202401878-s001.pdf]

ChemSusChem

Supporting Information

Rigid Plastic-Free Fibreboards Made from “Hairy” Cellulose Fibres and Oil Palm Empty Fruit Bunch

Dharu Feby Smaradhana, Diego Freire Ordóñez, and Koon-Yang Lee*

Supporting Information

Rigid Plastic-Free Fibreboards made from “Hairy” Cellulose Fibres and Oil Palm Empty Fruit Bunch

Dharu Feby Smaradhana, Diego Freire Ordóñez and Koon-Yang Lee

Table of Contents

S1. Experimental section 1
Materials 1
Refining of wood pulp using recirculating colloid mill 1
Manufacturing of EFB fibreboards using (hairy) cellulose fibres as the binders 1
Materials characterisation 2
Scanning Electron Microscope (SEM) 2
Porosity of EFB fibreboards 2
Tensile properties of EFB fibreboards 2
Internal bonding test 2
Flexural properties of EFB fibreboards 3
Tensile properties of (hairy) cellulose network 3
Apparent fibre pull-out strength between EFB fibre and (hairy) cellulose fibre network 3
Results of specific mechanical performances 4
S2. Lifecycle assessment analysis (LCA) 7
Goal and scope definitions 7
Lifecycle Inventory (LCI) 7
Business as usual scenario for EFB 8
LCA Inventory of EFB fibreboard using 30 wt.% 30 min refined hairy cellulose fibres as binder 11
LCA Inventory of medium density fibreboard (MDF) 12
Electricity mix profile in Indonesia (national average) and three different provinces in Indonesia 13
Lifecycle Impact Assessment (LCIA) 13
Uncertainty analysis 13
S3. Contribution distribution from EFB fibreboard production to GWP and endpoint categories 14
S4. Comparison of ReCiPe 2016 LCA results of various electricity sources in Indonesia 15
S5. Absolute uncertainties for MDF and EFB fibreboard 16
References 17

S1. Experimental section

Materials

EFB fibres were purchased from Polytech Indonesia in Karawang and originated from oil palm plants grown in West Kalimantan, Indonesia. As claimed by the supplier, the fibres were produced using shredding machines and were purified with 0.25 M NaOH under room temperature, rinsed with water, and sun-dried for a day. Upon receipt, the fibres were washed twice using DI water of 5 L/kg of EFB fibres and oven-dried at 80°C for two days before being cut into approximately 10 mm lengths. Acacia wood pulp was provided by Asia Pacific Resources International Limited (APRIL), Riau, Indonesia, in the form of pressed pulp sheets with a grammage of 500g/m^2. For comparison of flexural properties, commercial medium density fibreboard (MDF) of furniture grade and particleboard (PB) were purchased from a local retailer.

Refining of wood pulp using recirculating colloid mill

A pre-determined amount of pressed pulp sheet was cut into small pieces (around 1 cm × 1 cm). The small pulp sheets were then soaked into de-ionised water overnight. Afterwards, the pulp sheets were disintegrated using kitchen blender (Optimum 9400, Froothie Ltd., Cranleigh, UK) for 1 min with maximum power before fed into re-circulating colloid mill (JM-60, Shanghai Tiangang Machine Manufacture Co. Ltd., Shanghai, China) with cellulose pulp consistency of 1.5 wt%. The refining times used in this study were 10 mins, 20 mins and 30 min. Meanwhile, unrefined pulp suspension was prepared only using the kitchen blender.

Manufacturing of EFB fibreboards using (hairy) cellulose fibres as the binders

EFB fibreboards with grammage of 2000 g/m^2 containing 10 wt.%, 20 wt.%, or 30 wt.% of (hairy) cellulose fibres were manufactured. A pre-determined amount of dried EFB fibres was dispersed into cellulose pulp suspensions in 3.6 L of de-ionised water (~2.5 wt% of total solid content) and left soaking overnight. The suspensions were then vacuum filtered onto a 240 mm diameter filter paper (Qualitative filter paper 413, particle retention: 5–13 µm, VWR International Ltd., Lutterworth, UK) using a Büchner funnel. The wet filter cakes were sandwiched with two filter papers and two fresh blotting papers (Grade 3MM CHR, GE Healthcare, Buckinghamshire, UK) before wet pressed twice for 10 min (5 min for each wet pressing step) under a weight of 2 t. Finally, the wet pressed filter cakes were consolidated and dried using a hot press at 120°C under a weight of 2 t for 1 hour to produce EFB fibreboards. The manufactured EFB fibreboards using (hairy) cellulose fibres as the binder were stored in the sample bags containing dried silica gel until further characterisation.

Materials characterisation

Scanning Electron Microscope (SEM)

SEM (S-3700N, Hitachi High-Technologies Corporation, Tokyo, Japan) was conducted to characterise the surface of EFB fibreboards and the morphology of the constituents (EFB fibres and pulp fibres). An accelerating voltage of 15k V was used. Prior to SEM, all samples were fixed onto aluminium stubs using carbon tabs. Gold coating step was then performed using a sputter coater (Agar Auto Sputter coater, Agar Scientific Ltd., UK) for 40 s at 40 mA. In order to study the morphology of pulp fibres, low grammage papers (1 g/m^2) made from (un)refined pulp fibres were prepared. Suspension containing pre-determined unrefined or refined pulp with consistency of 0.05 wt% was vacuum filtered onto nylon fabrics (PP230 Nylon 66, Easy Composites Ltd, UK) with diameter of 125 mm using Büchner funnel. The wet filter cake was then air-dried and investigated using SEM following the previously described method.

Porosity of EFB fibreboards

The density of the manufactured fibreboards (ρ) was determined using Helium pycnometry (AccuPyc II 1340, Micrometrics Ltd., Dunstable, UK). Before placing the samples into the measuring chamber, the mass of the samples was measured. The envelope density (ρ_e) of the fibreboards was determined from their mass and envelope volume. The porosity (P) was calculated using measured ρ and ρ_e referring to equation (S1).

$$P = \left(1 - \frac{\rho_e}{\rho}\right) \times 100 \qquad \text{(S1)}$$

Tensile properties of EFB fibreboards

The tensile properties of EFB fibreboards were determined using universal testing machine equipment (Instron 5969, Instron GmbH, and Buckinghamshire, UK) equipped with 10 kN load-cell. The specimens were prepared by cutting fibreboards into dimensions of 100 × 15 × 3 mm using a laser cutter machine (Universal Laser Systems PLS6.75 laser platform, Scottsdale, United States). Prior to the test, 0.5 dots were marked on the surface of the specimens using a stamp (iMetrum Ltd., Bristol, UK). Strain was monitored and tracked based on the movement of the marks using a non-contacting video extensometer (iMetrum Ltd., Bristol, UK). Sandpapers with 180-grit were used as the end tabs of the specimens. The gauge length was 60 mm. The crosshead speed used was 1 mm/min. A minimum of 5 specimens was tested for each type of EFB fibreboards.

Internal bonding test

Specimens with dimension of 20 mm × 20 mm × 3 mm were prepared for internal bonding test. Before testing, the specimens were bonded with adhesive (Gorilla Glue, Buckshaw Village, UK) in between two wooden blocks and

left overnight to let the adhesive cure. Universal testing machine (Instron 5969, Instron GmbH, and Buckinghamshire, UK) with 10 kN load cell was used. The testing speed was 1 mm/min. A minimum of 5 specimens was tested for each type of EFB fibreboards.

Flexural properties of EFB fibreboards

Three-point bending test was performed to determine the flexural properties of EFB fibreboards using a universal testing machine (Instron 5969, Instron GmbH, and Buckinghamshire, UK) equipped with 10 kN load-cell. Prior to flexural testing, EFB fibreboards were cut into 80 × 15 × 3 mm using a laser cutter. The span length was 60 mm, and the crosshead speed was 2 mm/min. A minimum of 5 specimens was tested for each type of samples.

Tensile properties of (hairy) cellulose network

A sheet of paper depending on the refining times of (hairy) cellulose fibres with grammage of 300 g/m^2 were manufactured and characterised to confirm the effect of mechanical refining on the properties of the (hairy) cellulose fibres used as binder. After refining process, cellulose pulp suspensions with consistency of 0.25% were prepared and vacuum filtered onto 125 mm diameter filter paper (Qualitative filter paper 413, particle retention: 5–13 µm, VWR International Ltd., Lutterworth, UK) using a Büchner funnel. Afterwards, the wet cellulose pulp filter cake was sandwiched with two filter papers followed with two blotting papers (Grade 3MM CHR, GE Healthcare, Buckinghamshire, UK). The wet pressing under weight of 1 t was performed twice (5 mins for each press) with fresh blotting papers used for each pressing stage before hot pressing step under a weight of 1 t for 30 min at 120°C. The manufactured papers were stored in the sample bags with dried silica gel. Tensile test for (hairy) cellulose network was conducted using a micro-tensile tester (Model MT-200, Deben UK Ltd., Woolpit, UK) equipped with a 200 N load cell. A test specimen with dimension of 40 mm × 5 mm was used in this work. The specimens were secured and glued in between two paper frames (140 g/m^2) using a two-part cold curing epoxy resin (Araldite 2011, Huntsman Advanced Materials, UK). The gauge length was 25 mm. A non-contacting video extensometer (iMetrum Ltd., Bristol, UK) was used to record the strain of the specimens. The tensile test was conducted using 0.5 mm/min crosshead speed. A minimum of 5 specimens were tested for each type of samples.

Apparent fibre pull-out strength between EFB fibre and (hairy) cellulose fibre network

To assess how increasing the refining time of cellulose pulp affects the mechanical properties of EFB fibreboards, we investigated the fibre pull-out strength between EFB fibres and (hairy) cellulose fibre network using a single fibre-pull out test. This test involved embedding a single EFB fibre into a matrix created from cellulose pulp fibres refined for varying durations (0, 10, 20, or 30 minutes). We prepared a 300 ml of cellulose pulp suspension at a 0.25wt% consistency, poured onto a 55 mm diameter filter paper (Whatman, GE, United Kingdom), and used a Büchner funnel for vacuum filtration to achieve a filter cake with ~20% solid content. Five EFB fibres, each approximately 5 cm in length, were embedded 3-4 mm into the wet filter cake. These cakes were then sandwiched

between fresh filter papers and two blotting papers, followed by a pressing-drying stage at 120°C under a 1 ton weight for 30 minutes. The produced EFB fibre-embedded papers were cut into 10 mm × 20 mm strips, with each fibre centrally positioned. The actual embedded length of each fibre was measured by placing it over a light source. Afterwards, single fibre pull-out test was performed using a micro-tensile tester (Model MT-200, Deben UK Ltd., Woolpit, UK) equipped with a 200 N load cell. Prior to the test, each specimen was glued in between card paper frames (140 g/m^2) using epoxy resin (Araldite 2011, Huntsman Advanced Materials, UK). The crosshead speed used was 0.5 mm/min. A minimum of 10 specimens from each type of samples were tested. Apparent fibre pull-out strength is measured following equation (S2), where τ_i, l_e, and F_{deb} denote apparent fibre pull-out strength, embedded length and debonding force, respectively.

$$\tau_i = \frac{F_{deb}}{\text{Fibre perimeter} \times l_e} \quad \text{(S2)}$$

Results of specific mechanical performances

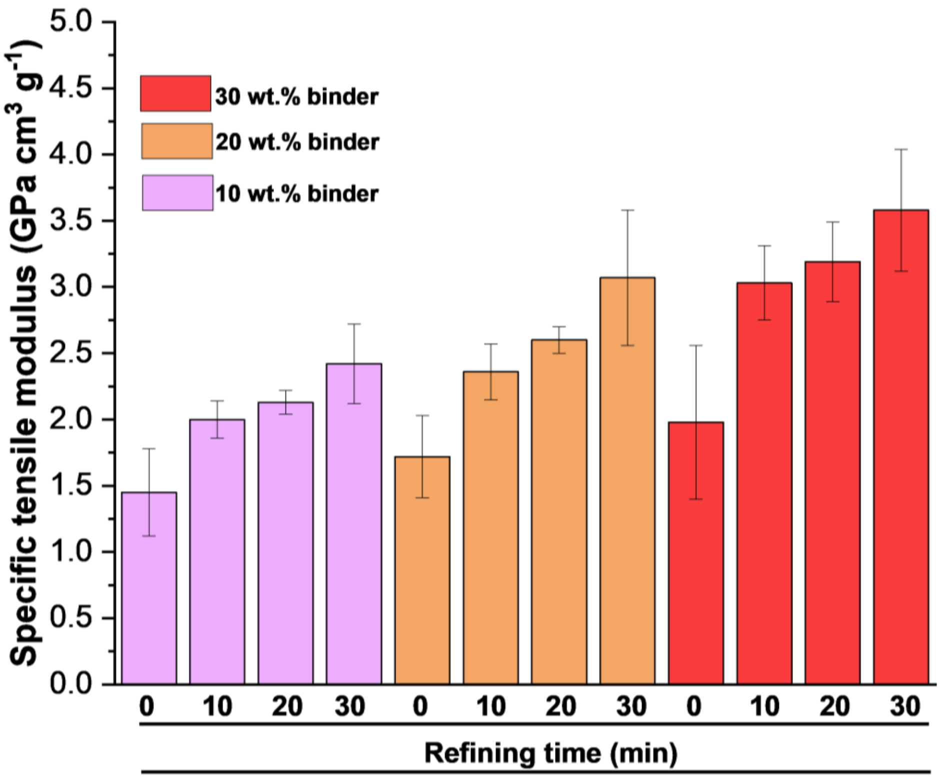

Figure S1. Specific tensile modulus of EFB fibreboards

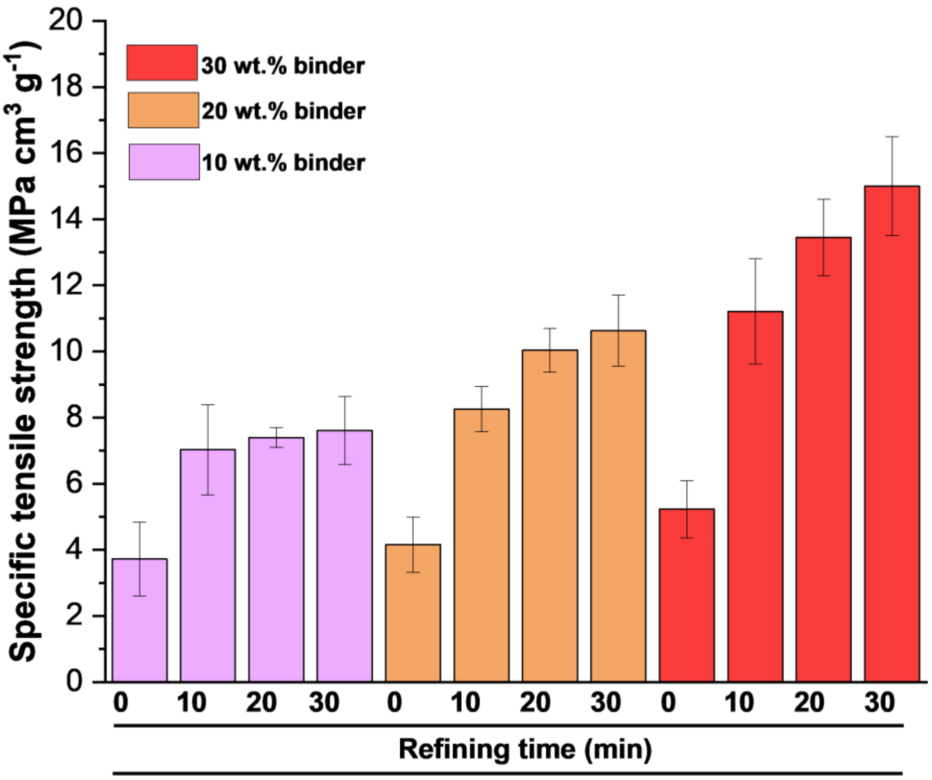

Figure S2. Specific tensile strength of EFB fibreboards

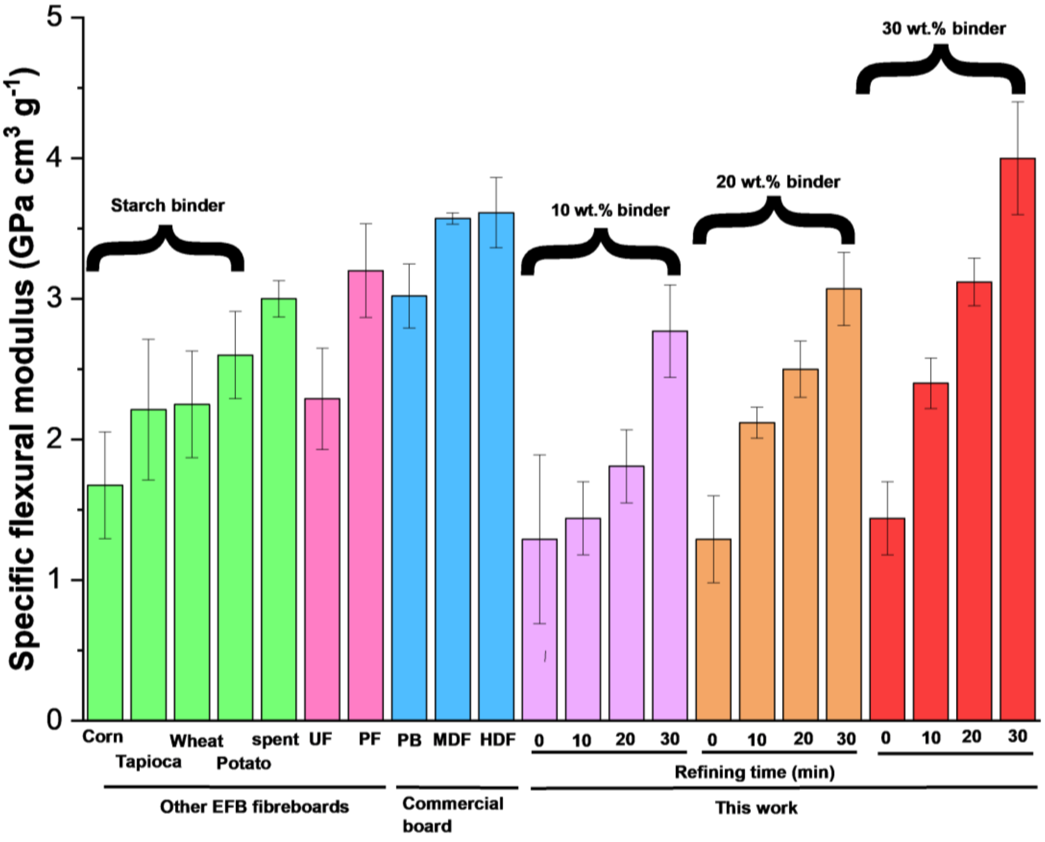

Figure S3. Specific flexural modulus of EFB fibreboards in this study and other fibreboards from literatures and commercial board.[1–5] UF, PF, PB, MDF and HDF denote urea formaldehyde, phenolic formaldehyde, particleboard, medium density fibreboard and high density fibreboard.

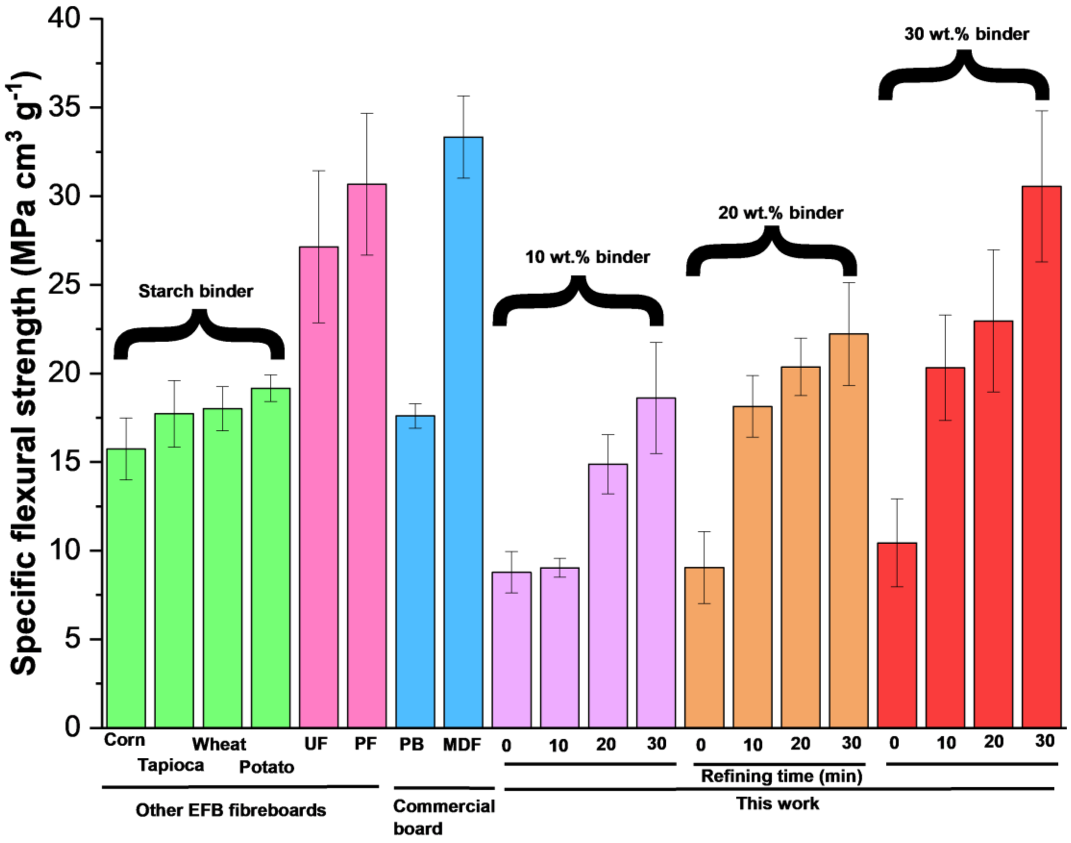

Figure S4. Specific flexural strength of EFB fibreboards in this study and other fibreboards from literatures and commercial board.[1–5] UF, PF, PB, MDF and HDF denote urea formaldehyde, phenolic formaldehyde, particleboard, medium density fibreboard and high density fibreboard.

S2. Lifecycle assessment analysis (LCA)

The environmental impact of the production of the EFB fibreboard was quantified using LCA following EN ISO 14040.[6] All calculations were carried out using SimaPro version 9.5 integrated with the Ecoinvent 3.8 database.

Goal and scope definitions

A cradle-to-gate approach was developed to include all lifecycle emissions from waste collection to the production of EFB fibreboards. The production of MDF is used as a benchmark for comparison. To compare materials with different mechanical performances, a performance indicator, specifically the specific flexural modulus, was employed. This indicator calculates the mass of the functional unit (m.fu) needed to match the flexural modulus level of an EFB fibreboard produced from 1 kg of EFB (40 wt% dry matter) and 30 wt% 30 min refined hairy cellulose fibres as the binder. The formula $m_{\mathrm{f.u.}}$ is provided in the equation (S3):

$$m_{\mathrm{f.u.}} = m_{\mathrm{ref}} \times \left[\frac{E_{\mathrm{ref}}}{E_{\mathrm{f.u.}}}\right]^{1/3} \left[\frac{\rho_{\mathrm{f.u.}}}{\rho_{\mathrm{ref}}}\right] \tag{S3}$$

Where m_{ref}, E_{ref}, and ρ_{ref} are the mass, flexural modulus and density of the EFB fibreboard respectively, while $m_{\mathrm{f.u.}}$, $E_{\mathrm{f.u.}}$, and $\rho_{\mathrm{f.u.}}$ are the mass, flexural modulus and density of the MDF counterpart respectively. The derivation of equation S3 can be found in the literature.[7]

Lifecycle Inventory (LCI)

The LCI combines data from the manufacturing process in this study (foreground system) and general data from the Ecoinvent v3.8 database (background system). An Allocation at the Point of Substitution (APOS) method was used to fully capture the environmental impacts of production and treatment processes.[8] The LCI for MDF counterpart is from the literature[9] with adjustment on the energy source for utilities. Both EFB fibreboard and MDF models utilise an energy source profile based on the Indonesia electricity grid (53% lignite, 21% combined cycle gas, 8% hydro, 5% natural gas, 5% oil, 3.5% biomass and others).[10] EFB fibreboard and MDF modelled in this LCA are assumed to be equally durable. It is assumed that in the manufacturing process of the EFB fibreboard, 90% of water was recycled to the system, with the remaining 10% disposed to the environment after wastewater treatment. The electricity usage of the recirculating colloid mill and the vacuum pump was measured using power metre (MECHEER, UK). The energy consumption for EFB fibre processing (shredding) was obtained from.[11] To estimate the energy required in drying steps, the following equation was used:

$$Q = \int_{T_1}^{T_2} \left(\sum_i m_i \times C_{P,i}\right) dT \tag{S4}$$

where Q is the energy required, T_1 and T_2 are the initial and final temperatures, m_i and $C_{P,i}$ are the mass and the heat capacity of compound i respectively. The heat capacity of EFB fibres and pulp fibres used as binder was 1.48 J/g K [12] and 1.4 J/g K [13] respectively. Biogenic CO_2 uptake (carbon stored in biomass) was attributed to both the

EFB fibreboards (including EFB and cellulose pulp as the binder) and MDF (wood fibres) since biomass can act as carbon sinks during its lifetime and can be used as carbon credits in the cradle-to-gate approach.[14] According to IPCC guidelines,[15] the carbon content of the biomass used in this study is assumed to be 50% of its dry weight. In business-as-usual scenarios, 50% of EFB was discarded on open land while the remaining portion was applied as mulching material reflecting the common practices conducted by smallholder farmers and industrial estates in Indonesia, respectively.[16] The detailed LCI inputs and parameters are provided below.

Business as usual scenario for EFB

Open discarding EFB

It is assumed that empty fruit bunches (EFB) are discarded on open area and unmanaged. The transport of waste to disposal area is included in the system boundary. Each EFB weighs approximately 5.75 kg, representing 23% of the weight of a fresh fruit bunch,[17] and has ellipsoid-like shape with a length of 600 mm and a width of 520 mm [17]. To dispose 1 ton of EFB (40% dry matter), it is assumed that an area of 7.9 m^2 is needed which accommodates 7 piles of EFB (~3.5 m deep) based on the dimension of EFB aforementioned above. This will transform an unspecified site into a dump site.

The estimation of methane (CH_4) generated in discarding 1 ton of EFB is evaluated based on IPCC guidelines for solid waste disposal (SWD), equation (S4) and (S5)[15].

$$Lo = W \, x \, DoC \times DoC_f \times MCF \times F \times \frac{16}{12} \tag{S4}$$

Where Lo, W, DoC, DoC_f, MCF and F are methane generation potential, mass of decomposable degradable organic carbon, the mass of waste, the fraction of degradable organic carbon, methane correction factor, and CH_4 concentration in landfill respectively*.* According to IPCC guidelines,[15] EFB, as agricultural waste or biomass, contains 50% carbon in dry matter or 20% in wet conditions (60% water content), validated by literatures[18,19] so that DoC for EFB in this work is set at 0.2 for 1 ton EFB with 60% water content. The DoC_f is set at 0.5 (default),[15] which indicates that half of the DoC in waste deposited at dump site converts into landfill gas, with the other half remaining in the dump site.[20] In order to determine Lo, the total amount of carbon decomposed is then multiplied by MCF of 0.4 (default) for unmanaged waste less than 5 metres deep[15], by F (set at 0.5 default) and by carbon content of CH_4 (16/12). The rest of carbon decomposed is assumed to be converted into CO_2, as unmanaged dump sites cannot create optimal anaerobic conditions and typically only 50% of the landfill gas consists of methane (F).[15,20] Table S1 shows the results from the equation (S4) for 1 ton EFB followed by table S2 showing the inventory for discarding 1 ton of EFB.

Table S1. Estimation of methane and carbon dioxide emissions from discarding 1 ton of EFB (40% dry matter) based on IPCC Guidelines[15]

**Category**	**Parameter**	**Value**	**Units**
**Inputs**	Mass of waste deposited (W)	1000	kg
	Degradable Organic Carbon (DoC)	0.2	fraction
	Fraction of DOC that can decompose (DoC_f)	0.5	fraction
	Methane Correction Factor (MCF)	0.4	fraction
	Fraction of CH_4 in generated gas (F)	0.5	fraction
**Results**	CH_4 generated (Lo)	26.7	kg
	CO_2 generated	293	kg

Table S2. Lifecycle inventory for open discarding 1-ton EFB (containing 40% dry matter)

**Products/Process**	Amount	Unit
Discarding EFB	1000	kg
**Condition**		
Transformation, from unspecified to dump site	7.9	m^2
**Inputs materials/processes**		
Transport from collection to dump site: freight, lorry	10	tkm
**Biogenic CO_2 Uptake (carbon stored)[1]**		
EFB	-730	kg
**Emission to air**		
CH_4	26.7	kg
CO_2	293	kg

[1]Carbon content of EFB is assumed to be 50% of its dry mass.[15] Biogenic CO_2 uptake of EFB is involved in this analysis to get the net value of Global Warming Potential [21,22]

Mulching EFB

EFB, when used as mulching materials, is presumed to undergo aerobic digestion because it is typically applied as a single layer of EFB.[23,24] Therefore, the biogenic CO_2 uptake in the EFB is assumed to effectively offset the direct emissions of CO_2 during aerobic decomposition. Furthermore, EFB as mulch also acts as an additional source of fertilizer in oil palm plantations and potentially reducing the number of chemical fertilizers used. According to literature,[25] 1 ton of EFB is equal to 6.1 kg of urea, 1.7 kg of Triple superphosphate and 16.3 kg of Potassium

Chloride. Consequently, the system expansion was carried out to demonstrate the production of fertilizer resulting from the mulching process. However, the increase in the use of chemical fertilizers was not significant when EFB was shifted from being used as mulch to being used for board panels. Additionally, oil palm plantations in Indonesia predominantly still depend on chemical fertilizers to nourish the soil. The transport from the collection of EFB to the oil palm plantation is considered according to literature.[23] EFB is usually applied surrounding the oil palm tree with application rate of 15 t/ha.[26]

Table S3. Lifecycle inventory for mulching 1 ton EFB (40% dry matter)

**Product**	Amount	Unit
Mulching EFB	1000	kg
**Avoided products**		
Chemical fertilizer:		
Urea[25]	6.1	kg
Triple superphosphate[25]	1.7	kg
Potassium Chloride[25]	16.3	kg
**Input materials/processes**		
Transport:		
Transport from collection to farm gate: lorry[23]	25	tkm
Tractor for applying EFB in the oil palm land: tractor and trailer, agricultural[23]	100	tkm
Subprocess:		
Mulching (15 t ha^{-1})[26]	0.067	ha

LCA Inventory of EFB fibreboard using 30 wt.% 30 min refined hairy cellulose fibres as binder

The EFB fibreboard factory is conveniently located just 10 km from the EFB collection site, allowing for the transport of 1 ton of EFB per trip.

Table S4. LCA inventory for production of EFB fibreboard (1 kg EFB h^{-1})

Product	Amount	Unit
EFB fibreboard (30 wt.% 30 min refined hairy cellulose fibres)	0.53	kg
**Avoided burdens**		
Business-as-usual scenario for EFB:		
Discarding EFB	0.5	kg
Mulching EFB	0.5	kg
**Input materials/processes**		
Fibre/fibre processing:		
EFB (40% dry matter)[1]	1	kg
Transport from collection to factory: lorry	0.1	tkm
NaOH (purification)	0.02	kg
HCl (dry weight)	0.018	kg
Binder:		
Kraft pulp	0.16	kg
Processing water:		
Water[2]	21.85	kg
**Input energy**		
Utilities – electricity, high voltage, Indonesia[3]:		
Shredding – EFB fibre[11]	0.07	kWh
Refining – hairy cellulose fibre binders	1.61	kWh
Vacuum filtration	0.04	kWh
Pressing and drying	0.29	kWh
**Biogenic CO_2 uptake (carbon stored)[4]**		
EFB fibre	0.69	kg
Cellulose pulp binder	0.29	kg
**Emission to air**		
Water (vapour)	0.35	kg
**Output to technosphere: Waste treatment**		
Wastewater, treatment of wastewater[1]	2.15	kg

[1]after purification, 0.37 kg of clean EFB fibres are recovered from 0.4 kg of dry EFB fibres, indicating a 7.5% loss during the process.
[2]it is assumed 10% of water is sent to wastewater treatment and 90% recirculated to the process.
[3]see table S6 for the details on electricity grid profile for Indonesia.
[4]carbon content in EFB fibre and cellulose pulp binders is assumed to be 50% of the dry mass[15]

LCA Inventory of medium density fibreboard (MDF)

Table S5. LCA Inventory production of 1 m^3 MDF from[9]

**Product**	Amount	Unit
MDF	741	kg
**Input materials/processes**		
Fibre/fibre processing:		
**Wood fibres:**		
Wood chips	455	kg
Sawdust	151	kg
Shavings	187	kg
Binder:		
Urea-formaldehyde resin	83.3	kg
Paraffin, Wax	5.21	kg
Urea scavenger	1.28	kg
Processing water:		
Water	1387	kg
**Input energy**		
Utilities – electricity, high voltage, indonesia[1]:		
Heating	2552.24	kWh
Equipment	414.73	kWh
**Biogenic CO_2 uptake (carbon stored)**		
wood[2]	1239	kg
**Emission to air[3]**		
Nitrogen oxides	0.38	kg
Sulfur oxides	0.0073	kg
VOC	0.84	kg
Particulate	0.36	kg
Particulate (PM10)	0.29	kg
Formaldehyde	0.16	kg
Methanol	0.22	kg
**Emission to water**		
Suspended solids	0.01	kg
BOD	0.0072	kg
Ammonia nitrogen	0.0023	kg
**Output to technosphere: Waste treatment**		
Wood ash mixture, treatment of, sanitary landfill	1.94	kg
Waste wood, treatment of, sanitary landfill	2.21	kg

[1]the energy source for utilities is modified to only use electricity, high voltage, Indonesia.
[2]this MDF consists of 10% binder and 90% of wood chips. The biogenic CO_2 is obtained from the carbon stored in the wood chips – 50% of its dry weight.[15]
[3]direct emissions derived from fuel i.e. natural gas, wood fuel and oil (from the original inventory[9]) were excluded in this inventory since the energy used is only electricity.

Electricity mix profile in Indonesia (national average) and three different provinces in Indonesia

This electricity grid profile was modelled based on the most recent report by Indonesian Ministry of Energy and Mineral Resources (ESDM) in 2022.[10] The background data is the production of high voltage electricity in Indonesia provided in Ecoinvent.

Table S6. Electricity mix profile in Indonesia

Source	National average (%)	North Sumatra (%)	Riau (%)	West Kalimantan (%)
Lignite	53.16	24.93	14.07	40.44
Natural gas	5.32	4.12	2.74	3.15
Cycle gas	21.28	26.02	22.74	9.27
Hydro	7.98	26.46	4.44	21.92
Biomass	3.51	3.33	48.44	3.62
Biogas	0.22	0.49	1.20	0.19
Oil	5.19	3.11	6.36	21.22
Wind	0.18	-	-	-
Solar	0.34	0.01	-	0.18
Deep geothermal	2.82	11.53	-	-

Lifecycle Impact Assessment (LCIA)

The ReCiPe 2016 method,[27] following a hierarchist perspective[28], is utilised as implemented in SimaPro 9.5. The endpoint indicators aggregate various midpoint indicators as follows:

- **Human health (DALY)** includes impacts from global warming, stratospheric ozone depletion, ionising radiation, ozone formation, fine particulate matter formation, carcinogenic and non-carcinogenic toxicity, and water consumption affecting human health.
- **Ecosystems (species.yr)** accounts for the effects of global warming on terrestrial and freshwater ecosystems, ozone formation, acidification, eutrophication across environments, ecotoxicity, land use, and water consumption on both terrestrial and aquatic ecosystems.
- **Resources (USD_{2013})** evaluates the scarcity of mineral and fossil resources.

Uncertainty analysis

A Monte Carlo simulation was conducted to analyse the uncertainty within LCI data evaluating all midpoint and endpoint indicators using SimaPro. This method involves random sampling to model data variability through probability distributions, with each uncertain parameter undergoing 1000 iterations with confidence intervals of 95%.[29] The Pedigree Matrix, which evaluates data on criteria such as reliability and completeness, is used to determine the parameters of the underlying lognormal distributions that model the LCI elementary flows like feedstock, emissions, and waste.[30]

S3. Contribution distribution from EFB fibreboard production to GWP and endpoint categories

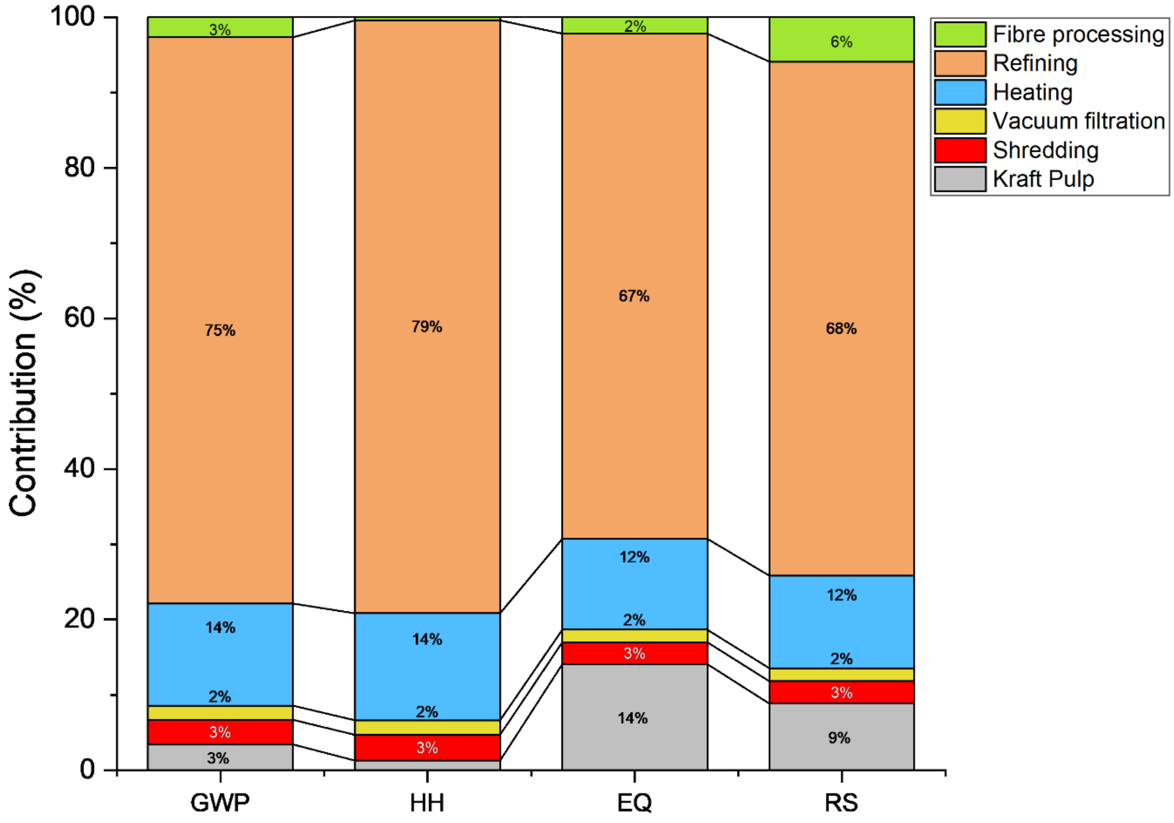

Figure S5. Contribution distribution for GWP and three endpoint categories to produce EFB fibreboard. The percentages of contributions lower than 1%are not indicated.

S4. Comparison of ReCiPe 2016 LCA results of various electricity sources in Indonesia

Table S7. ReCiPe 2016 LCA results of various electricity sources (high voltage) in Indonesia for producing 1 kWh

Damage category	Unit	Lignite	Natural gas	Combined cycle gas	Hydro	Biomass	Biogas	Oil	Deep geothermal
Human health	DALY	2.67E-05	9.64E-07	5.59E-07	8.12E-08	5.26E-07	5.32E-07	2.75E-06	1.49E-07
Ecosystems	species.yr	7.80E-09	2.80E-09	1.62E-09	3.36E-10	6.81E-09	2.97E-09	5.88E-09	3.07E-10
Resources	USD2013	0.002441946	0.11848047	0.070045381	0.000348665	0.006200953	0.008982342	0.15658776	0.0033592

S5. Absolute uncertainties for MDF and EFB fibreboard

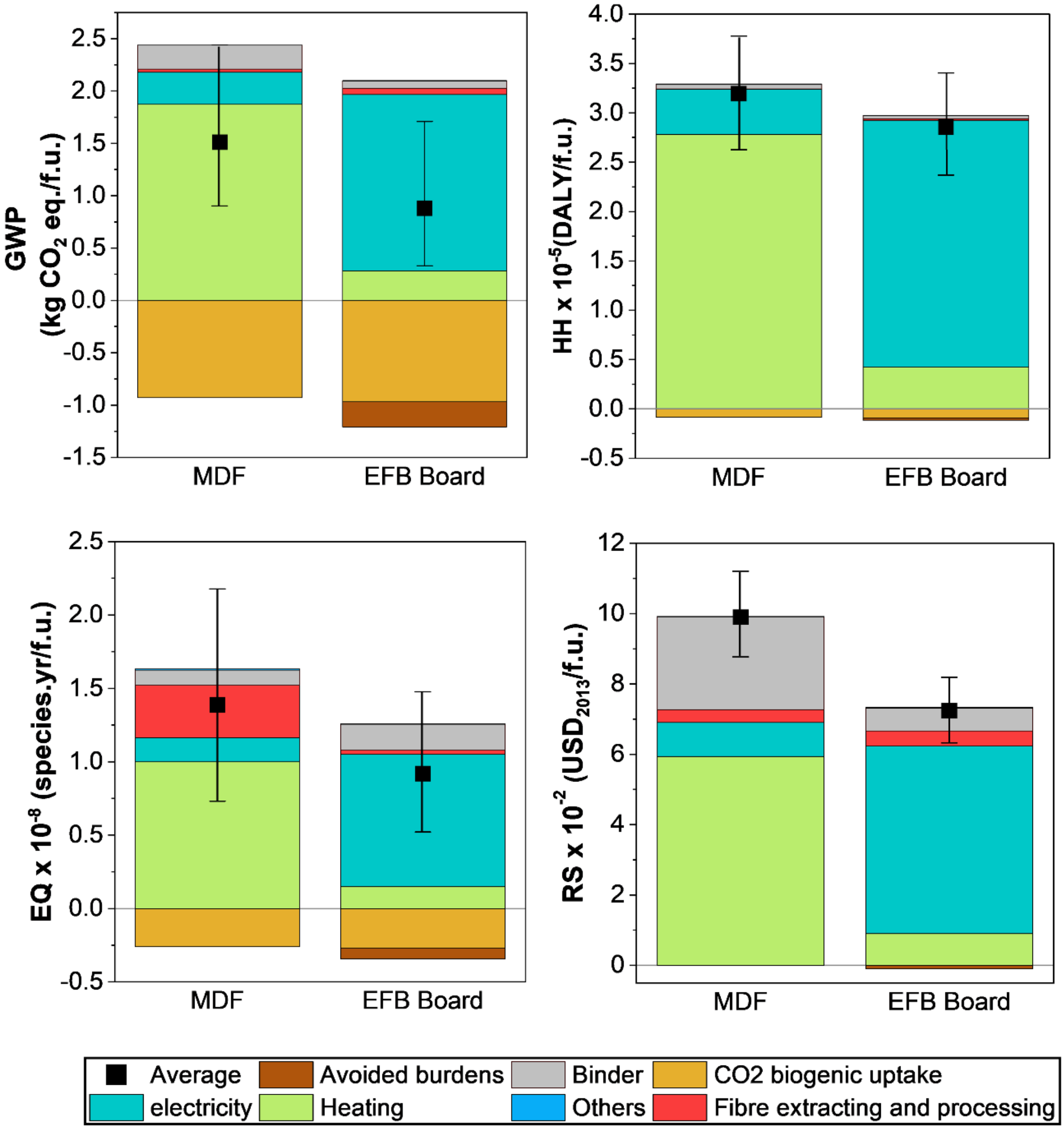

Figure S6. ReCiPe 2016 LCA results at the endpoint level and global warming potential for MDF and EFB fibreboard with absolute uncertainties (error bars indicate the 95% CI for the mean).

References

[1] H. P. S. Abdul Khalil, A. M. Issam, M. T. Ahmad Shakri, R. Suriani, A. Y. Awang, *Industrial Crops and Products* **2007**, *26*, 315–323.
[2] D. Theng, G. Arbat, M. Delgado-Aguilar, F. Vilaseca, B. Ngo, P. Mutjé, *Industrial Crops and Products* **2015**, *76*, 166–173.
[3] R. Wahab, M. S. M. Rasat, H. W. Samsi, M. T. Mustafa, S. M. M. Don, *JAS* **2017**, *9*, 237.
[4] W. N. A. W. Nadhari, N. S. Ishak, M. Danish, S. Atan, A. Mustapha, N. A. Karim, R. Hashim, O. Sulaiman, A. N. A. Yahaya, *Materials Today: Proceedings* **2020**, *31*, 287–291.
[5] M. A. Shakir, M. I. Ahmad, F. Z. Mansur, N. K. Ramli, S. A. Zaki, *Waste Biomass Valor* **2024**, DOI 10.1007/s12649-024-02453-z.
[6] M. Finkbeiner, A. Inaba, R. Tan, K. Christiansen, H.-J. Klüppel, *Int J Life Cycle Assessment* **2006**, *11*, 80–85.
[7] A. N. Gaduan, K. Singkronart, C. Bell, E. Tierney, C. Burgstaller, K.-Y. Lee, *ACS Appl. Polym. Mater.* **2022**, *4*, 3294–3303.
[8] G. Wernet, C. Bauer, B. Steubing, J. Reinhard, E. Moreno-Ruiz, B. Weidema, *Int J Life Cycle Assess* **2016**, *21*, 1218–1230.
[9] J. B. Wilson, *WOOD AND FIBER SCIENCE* **2010**, *42*.
[10] Sansuadi, Nur Mazidah, Rahmad Cahyo Nugroho, Nur Hidayanto, *Statistik Ketenagalistrikan 2022*, Sekretariat Direktorat Jenderal Ketenagalistrikan, **2023**.
[11] D. K. S. Ng, R. R. Tan, D. C. Y. Foo, M. M. El-Halwagi, Eds. , *Process Design Strategies for Biomass Conversion Systems*, Wiley, **2015**.
[12] B. B. Nyakuma, A. Johari, A. Ahmad, T. A. T. Abdullah, *Energy Procedia* **2014**, *52*, 466–473.
[13] The Engineering Toolbox, “Specific Heat of common Substances,” can be found under https://www.engineeringtoolbox.com/specific-heat-capacity-d_391.html, **2023**.
[14] D. Ita-Nagy, I. Vázquez-Rowe, R. Kahhat, I. Quispe, G. Chinga-Carrasco, N. M. Clauser, M. C. Area, *Science of The Total Environment* **2020**, *720*, 137586.
[15] the National Greenhouse Gas Inventories Programme, Eggleston H.S., Buendia L., Miwa K., Ngara T. and Tanabe K., *2006 IPCC Guidelines for National Greenhouse Gas Inventories*, IGES, Japan, **2006**.
[16] K. Rudolf, N. Hennings, M. A. Dippold, E. Edison, M. Wollni, *Agricultural Systems* **2021**, *193*, 103242.
[17] N. Jungbluth, N., Chudacoff, M., Dauriat, A., Dinkel, F., Doka, G., Faist Emmenegger, M., Gnansounou, E., Kljun, N., Schleiss, K., Spielmann, M., Stettler, C., Sutter, J., *Life Cycle Inventories of Bioenergy. Ecoinvent Report No. 17*, Swiss Centre For Life Cycle Inventories, Dübendorf, **2007**.
[18] M. A. A. Mohammed, A. Salmiaton, W. A. K. G. Wan Azlina, M. S. Mohamad Amran, *Bioresource Technology* **2012**, *110*, 628–636.
[19] S. H. Chang, *Biomass and Bioenergy* **2014**, *62*, 174–181.
[20] U. Lee, J. Han, M. Wang, *Journal of Cleaner Production* **2017**, *166*, 335–342.
[21] A. Levasseur, P. Lesage, M. Margni, R. Samson, *J of Industrial Ecology* **2013**, *17*, 117–128.
[22] A. Downie, D. Lau, A. Cowie, P. Munroe, *Biomass and Bioenergy* **2014**, *60*, 18–31.
[23] E. I. Wiloso, C. Bessou, R. Heijungs, *Int J Life Cycle Assess* **2015**, *20*, 204–216.
[24] A. S. Putra, R. Noguchi, T. Ahamed, A. Nakagawa-Izumi, H. Ohi, *Int J Life Cycle Assess* **2021**, *26*, 175–188.
[25] I. Comte, F. Colin, O. Grünberger, S. Follain, J. K. Whalen, J.-P. Caliman, *Agriculture, Ecosystems & Environment* **2013**, *169*, 58–68.
[26] T. D. Alcock, D. E. Salt, P. Wilson, S. J. Ramsden, *Science of The Total Environment* **2022**, *829*, 154539.
[27] M. A. J. Huijbregts, Z. J. N. Steinmann, P. M. F. Elshout, G. Stam, F. Verones, M. Vieira, M. Zijp, A. Hollander, R. van Zelm, *Int J Life Cycle Assess* **2017**, *22*, 138–147.
[28] M. Tomatis, M. T. Moreira, H. Xu, W. Deng, J. He, A. M. Parvez, *Journal of Cleaner Production* **2019**, *233*, 808–818.
[29] Mark Goedkoop, Michiel Oele, Marisa Vieira, Jorrit Leijting, Tommie Ponsioen, Ellen Meijer, *SimaPro Tutorial*, PRé, **2016**.
[30] A. Ciroth, S. Muller, B. Weidema, P. Lesage, *Int J Life Cycle Assess* **2016**, *21*, 1338–1348.
